# The Effects of Sleeve Gastrectomy on the Appetitive Domain of Taste Using the Progressive Ratio Task

**DOI:** 10.1007/s11695-023-07035-x

**Published:** 2024-02-19

**Authors:** Noura K. Althukair, Ghalia N. Abdeen, Carel W. le Roux, Alex D. Miras, Aayed R. Alqahtani

**Affiliations:** 1 Department of Community Health Sciences, College of Applied Medical Sciences, King Saud University, Riyadh, Saudi Arabia; 2 Division of Diabetes, Endocrinology and Metabolism, Imperial College London, 5, London, UK; 3 Diabetes Complications Research Centre, Conway Institute, University College Dublin, 10, Dublin, Ireland; 4 Gastrosurgical Laboratory, University of Gothenburg, Gothenburg, Sweden; 5 Department of Surgery, College of Medicine, King Saud University, Riyadh, Saudi Arabia

**Keywords:** Obesity, Sleeve gastrectomy, Appetitive reward, Taste, Ingestive behaviour, Mechanism

## Abstract

**Introduction:**

Sleeve gastrectomy (SG) is an effective treatment for obesity in adolescents. The underlying weight loss mechanism may impact the peripheral and central gustatory system along with reward circuits in the brain. This study aims to assess changes in appetitive behavior in short-, medium-, and long-term follow-up.

**Methods:**

In this prospective observational study, a total of 8 adolescents with obesity who underwent SG and 9 comparator unoperated participants were studied. Appetitive behaviour towards fat and sweet taste stimuli was assessed using the Progressive Ratio Task (PRT) over a 6 year period.

**Results:**

Mean body mass index (BMI) of the surgical patients dropped from 51.5 ± 2.8 kg/m^2^ to 31.4 ± 1.9 and 30.9 ± 2.3 kg/m^2^ at 1 and 6 years follow-up, respectively. (*p* < 0.001). The median (interquartile range) total rewards earned during the PRT was 6 (5–7) pre-surgery, 5 (3–6) after one year and 4 (2–4) after six years from surgery (p = 0.007).

**Conclusion:**

SG reduced appetitive behaviour at 1 year with maintained the benefit over 6 years as measured by the progressive ratio task.

**Graphical Abstract:**

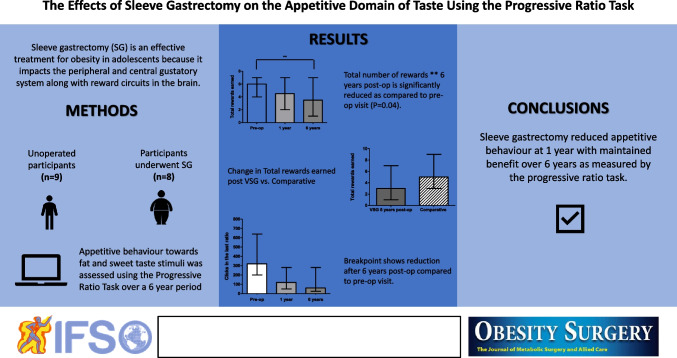

## Introduction

Obesity is a complex and chronic disease that results in increased appetitive behaviour and food intake causing an accumulation of adipose tissue [1]. An effective treatment for obesity in adults and adolescents is sleeve gastrectomy [2–4]. Children and adolescents after 2 years of sleeve gastrectomy show successful weight loss, resolution of obesity complications, and unaltered growth. Patient continue to improve for at least 10 years [5, 6].

Food intake is a consequence of behaviour and not necessarily a behaviour in its own right. Ingestive behaviours are impacted by the sensory domain of taste, the reward domain of taste, and the physiological domain of taste [7]. The palatability of the stimulus directly impactsfood reward [7].

The underlying mechanisms causing weight loss after sleeve gastrectomy includes changes in the peripheral and central gustatory system [8] along with reward circuits in the brain [9]. Patients change their eating behaviour after sleeve gastrectomy [10–13], with alteration in the sensory and reward domains of taste for calorie dense foods. Gastric bypass surgery also has an effect on appetitive behavior, and results in fewer calories being consumed [14–17].

The progressive ratio task, developed by Hodos, is the gold standard for assessing appetitive reward in both animals and humans [19]. The motivation to obtain a reward such as food (appetitive behaviour) was assessed in both animals and humans after bariatric surgery using the progressive ratio task [9, 14, 17, 18], but no data beyond one year exist. The aim of the present study was to assess the appetitive behavior after SG in in adolescents pre-surgery, 1, and 6 years post-operation.

## Materials and Methods

This prospective study collected data from adolescents undergoing SG performed by a single surgeon using a standardized technique [4]. Children and adolescents were eligible for SG if they satisfied the following inclusion criteria (body mass Index (BMI) ≥ 40 kg/m^2^, or ≥ 35 kg/m^2^ with complications). A comparator group of the same age was recruited from the community. The comparator group did not have any weight loss interventions. Exclusion criteria included pregnancy, breast feeding, a diagnosis of type 2 diabetes or psychiatric illness, dental issues, and the inability to understand instructions. Written, informed consent was obtained from all the participants. The study was approved by the local Ethics Committee (Reference E 19–3628).

During the Progressive Ratio Task (PRT), the subjects press a button in a geometric progressive ratio (10, 20, 40, 80, 160, 320, 640, etc.…) to obtain a reinforcer (i.e. reward). The subject continue to receive a reward until such time when the effort of pressing the button exceeds the value of the reward for the subject. This point is defined as the breakpoint. The number of clicks “in the last completed ratio” indicates the reward value of the reinforcer and this task is a pure assessment of appetitive responsiveness driven by the stimulus properties of the reinforcer, such as its taste. The benefit of this approach is the very low calorific content of the reinforces, thus reducing postingestive feedback.

All participants were instructed to have their usual breakfast until they felt “comfortably full” before they came to the facility. Testing occurred 2–3 h after breakfast in a quiet room within the clinical research facility. The room temperature was maintained at 21 °C. Participants were blinded to the study hypothesis, given exactly the same verbal and written instruction and specifically informed that there were no right or wrong responses to the task.

A power point presentation in Arabic explained the PRT program and provided instructions. Participants rated their hunger, fullness, desire to eat, and nausea immediately prior to and after the test using a horizontal 100 mm Visual Analogue Scale (VAS) with the anchors “not at all” and “extremely” on either end. Participants were placed in front of a computer screen and a plate of 20 chocolate candies (M&M® crispy candies, Mars UK Limited, Slough UK), each one containing approximately 4 kcal (energy contribution: 43.7% sugars, 44.1% fat). The following prompt appeared on the screen: “You can earn food by clicking on the mouse button. Click as much or as little as you like. When you no longer want to continue, press the spacebar to stop the session”. Upon completion of each ratio a message box appeared on the screen: “You have earned food. Enjoy your reward and after you have swallowed it completely you may click on OK to continue with the program.” After ingesting the reward, the participants then pressed the OK button in the message box only if they wished to progress to the next ratio in order to obtain another chocolate candy. The geometric progression schedule was chosen based on previous experiments within our research group [14].

The instructor ensured that all participants understood the experiment, and then left the room and participants were left on their own to complete the task. The instructor was not present during the task to reduce any potential influence on the behavioral responses of the participant [14]. When the effort of repeatedly pressing the mouse button was perceived to be greater than the reward value of the chocolate candy, participants pressed on the space bar to terminate the session indicating the breakpoint was reached. No food or fluid was offered after termination. The same numbers of chocolate candies (n = 20) were presented to all participants. The number of candies left after completion of the experiment was subtracted from 20 to give the total number consumed. This was correlated with the number of completed ratios from the computer software to ensure participants followed the instructions. The sample size was based on similar studies which previously showed significant results with 9–15 participants after Roux-en-Y Gastric Bypass (RYGB) [14].

Comparisons between and within groups were made using the Mann-Witney and Wilcoxon matched pairs test respectively. One-way ANOVA, within the surgical group was performed using repeated measures, Bonferroni and the Friedman test respectively. Correlations were done using the Spearman non parametric test, but the graphs include a parametric linear regression curve for visual comparison. The patient characteristic data were normally distributed and thus t-tests and ANOVA were used for within and between group comparisons for age and BMI, whilst gender comparisons were made with Fisher’s exact test. Results were expressed either as mean ± SEM or median (interquartile range). GraphPad Prism® version 5 was used for statistical comparisons.

## Results

All eligible non-surgical participants in the comparator group completed the study (n = 9). The mean age of the comparator group was 20.6 ± 0.5 years. In the surgical group 16 participants who were studied initially were contacted 6 years after surgery. Three participants were unreachable, two participants were studying abroad, and three refused to participate in this follow-up study. A total of eight participants from the SG group with a mean age of 21.8 ± 0.6 years were included and completed the study (Table 1).

**Table 1:** Demographic characteristics of the participants

	VSG 6 years post-op (N = 8)	Matched control group (N = 9)	*P* value Between Groups
Participants characteristics			
Gender (M/F)	4/4	3/6	
Age (years)	21.8 ± 0.6	20.6 ± 0.5	N/A
Weight (kg)	81.6 ± 6.9	59 ± 2.9	0.007*
BMI (kg/m^2^)	30.9 ± 2.3	22.0 ± 0.6	0.002*
Response at the PRT			
Clicks in the last ratio	60 (25–280)	160.0 (80–960)	0.1
Total rewards earned	4 (2–6)	5 (4–7)	0.1
Total clicks	131 (10–1270)	372 (76–9789)	0.21
Visual analogue scale rating			
Hunger pre-test (mm)	18.1 ± 5.2	38.5 ± 9.6	0.1
Fullness pre-test (mm)	64.6 ± 11.1	58.2 ± 8.7	0.7
Wanting pre-test (mm)	36.1 ± 8.7	30.0 ± 7.8	0.6
Nausea pre-test (mm)	29.8 ± 12.7	17.3 ± 8.3	0.4
Hunger post-test (mm)	10.2 ± 5.6	32.1 ± 11.0	0.1
Sweetness post-test (mm)	72.8 ± 10.0	80.8 ± 6.2	0.5
Creaminess post-test (mm)	71.0 ± 10.5	55.0 ± 9.9	0.3
Liking post-test (mm)	40.8 ± 12.4	65.7 ± 7.3	0.1
Nausea post-test (mm)	50.3 ± 14.2	17.8 ± 8.5	0.1

N number of patients; BMI body mass index; PRT progressive ratio task; VAS visual analogue scale ratings. Results are expressed as mean ± SEM or median (interquartile range) depending on normality distribution

* *p*-value is significant

For the intervention group, the BMI pre surgery was 51.5 ± 2.8 kg/m^2^, but then reduced after SG and remained stable between 1 and 6 years, 31.4 ± 1.9 vs 30.9 ± 2.3 kg/m^2^ (*p* < 0.001). The percentage of excess weight loss (%EWL) was 68.6 ± 4.7% after 1 year and 72.9 ± 4.7% after 6 years. The mean BMI of the comparator group was 22.0 ± 0.6 kg/m^2^.

**Fig. 1:**
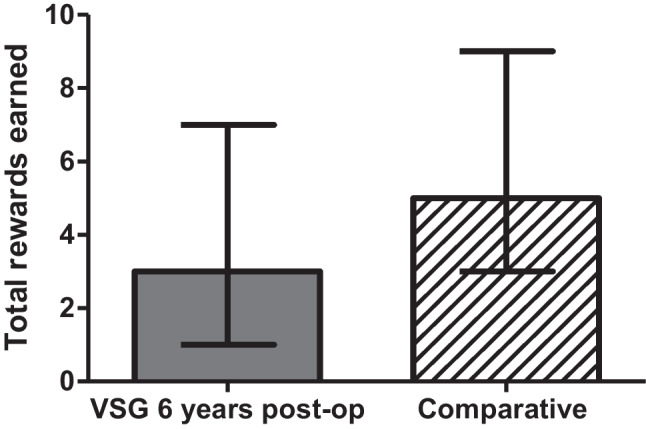
Change in Total rewards earned post VSG vs. Comparative. Graph plot of the total Number of rewards for M&M® crispies, there were no significant differences in total rewards eardned between 6 years post-op in VSG group with comparative group

**Fig. 2:**
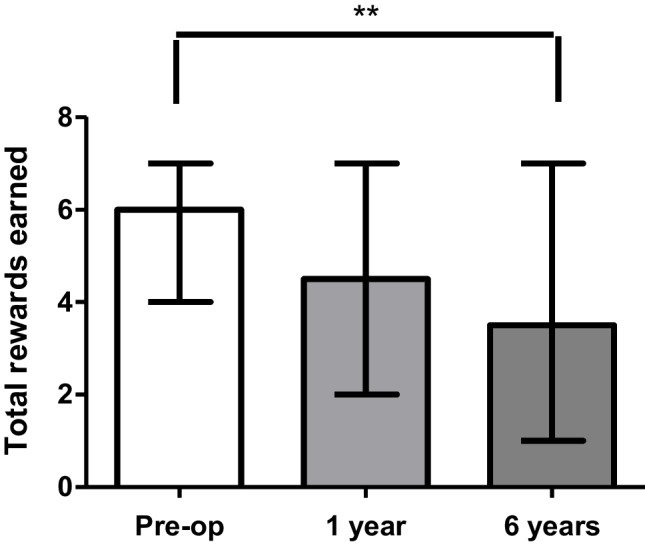
Change in Total rewards earned pre/post VSG. Graph plot of the total Number of rewards for M&M® crispies, ** 6 years post-op is significantly reduced as compared to pre-op visit (*P* = 0.04)

**Fig. 3:**
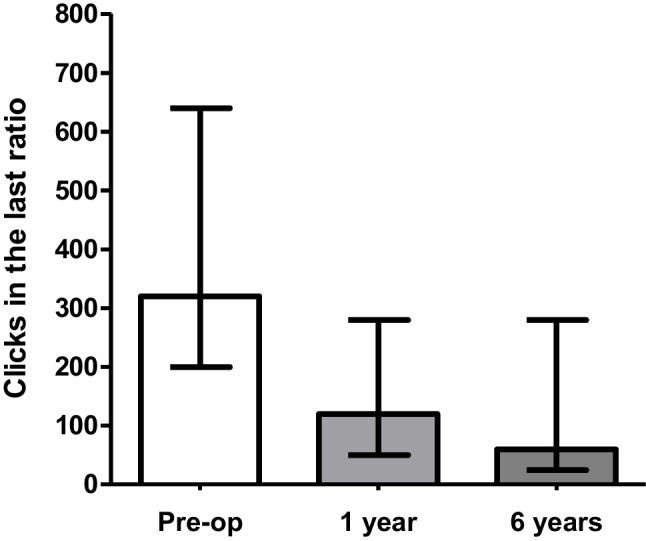
Change in clicks in the last ratio pre/post VSG. Graph plot of breakpoint (i.e. Clicks in the last completed ratio) for M&M® crispies. Shows reduction after 6 years post-op compared to pre-op visit

There were no discrepancies between the numbers of reinforcers consumed (counted after the session) and the number predicted to have been consumed based on the software results for either the SG or comparator groups (Fig. 1). For the comparator group the median (interquartile range) total rewards earned during the PRT was 5 (4–7). For the SG group the median (interquartile range) total rewards earned during the PRT was 6 (5–7) pre-surgery, 5 (3–6) after one year and 4 (2–4) after six years from surgery (*p* = 0.007) (Fig. 2). The median breakpoint during the PRT was 320 (200–640) pre-surgery, 120 (50–280) after 1 year and 60 (25–280) after 6 years (*p* = 0.07) (Fig. 3) (Table 2). There was no correlation in between changes in weight and total clicks at 6 years post SG (*p* = 0.38). There were no differences observed in VAS results between SG group at all visits (*P* > 0.05). There was no difference in the number of rewards consumed between the comparator group and the SG at 6 years (*p* = 0.1). Similarly, no differences were shown between the VAS results for the SG and comparator groups at 6 years follow-up.

**Table 2:** One Way ANOVA results characteristics for VSG group at pre-op, 1 years and 6 years post-op

	VSG Pre-op (N = 8)	VSG 1 year Post-op (N = 8)	VSG 6 years Post-op (N = 8)	*P* value Within VSG groups
Participants characteristics				
Gender (M/F)	4/4	4/4	4/4	
Age (years)	14.6 ± 0.6		21.8 ± 0.6	< 0.0001*
Weight (kg)	135.5 ± 9.0	84.4 ± 6.9	81.6 ± 6.9	< 0.0001*
BMI (kg/m^2^)	51.5 ± 2.8	31.4 ± 1.9	30.9 ± 2.3	< 0.0001*
%EWL (%)		68.6 ± 4.7	72.9 ± 4.7	
Response at the PRT				
Clicks in the last ratio	320 (200–640)	120 (50–280)	60 (25–280)	0.07
Total rewards earned	6 (5–7)	5 (3–6)	4 (2–6)	0.007*
Total clicks	896 (150–1292)	230 (10–1270)	131 (10–1270)	0.29
Visual analogue scale rating				
Hunger pre-test (mm)	39.3 ± 11.07	33.6 ± 10.8	18.1 ± 5.2	0.4
Fullness pre-test (mm)	55.1 ± 10.7	66.0 ± 10.5	64.6 ± 11.1	0.8
Wanting pre-test (mm)	55.3 ± 11.1	39.4 ± 8.4	36.1 ± 8.7	0.1
Nausea pre-test (mm)	14.5 ± 7.8	27.8 ± 13.0	29.8 ± 12.7	0.4
Hunger post-test (mm)	22.3 ± 11.0	25.0 ± 9.3	10.2 ± 5.6	0.7
Sweetness post-test (mm)	77.5 ± 9.5	72.5 ± 10.7	72.8 ± 10.0	0.9
Creaminess post-test (mm)	62.4 ± 12.9	74.5 ± 6.9	71.0 ± 10.5	0.8
Liking post-test (mm)	59.5 ± 14.2	45.4 ± 12.0	40.8 ± 12.4	0.6
Nausea post-test (mm)	30.1 ± 10.5	39.9 ± 13.5	50.3 ± 14.2	0.1

N number of patients; BMI body mass index; %EWL percentage of excess weight loss; PRT progressive ratio task; VAS visual analogue scale ratings. Results are expressed as mean ± SEM or median (interquartile range) depending on normality distribution

* *p*-value is significant

## Discussion

Sleeve Gastrectomy reduces weight and appetitive behaviour in adolescents at 1 year and benefits were maintained over 6 years. The findings are consistent with studies conducted after RYGB in 1) humans followed for 8 weeks [14], in 2) rats post SG followed for 8 weeks [18] and in 3) humans after SG for 12 months [20]. In contrast, other studies showed an increase in the appetitive behaviour after 6 months post RYBG in both rats [9] and humans [17]. One of the possible explanations for the discrepancy is that these studies used differed methodologies to test appetitive behavior. It is also possible that appetitive behaviour may be different in the weight loss phase (most negative energy balance in first 3–6 months) and the weight stable phase after 12 months.

Depending on the stimulus and the reward system, the responses can be either appetitive or aversive. People usually tend to ingest more food when the food is pleasant, regardless of the different taste qualities [22]. Appetitive behavior is a taste related motivational component that represents the willingness of a subject to work to obtain a reward, in this case food [21].

We used the same protocol for PRT as previously [14, 20]. PRT measures behaviour directly and is not burdened by interpretive limitations associated with scaling procedures or verbal report. Moreover, the method uses simple computer software, in which participants tasted the reinforcer during the task rather than postponing the consumption to the end. Furthermore, appetitive responsiveness did not rely on the association between a stimulus like a token, money or images with the reward, instead it was determined directly by the orosensory properties (e.g. taste) of the reward. Furthermore, in order to minimize bias in the responses, the investigator was absent from the room during the test.

The number of rewards earned by the SG group at 6 years decreased as compared to the pre-op visit and was stable compared to 1-year post-op visit. The number of mouse clicks in the last completed ratio by the SG group after 6 years showed a trend to decrease compared with pre-op visit, while being similar when compared to 1-year after surgery. The variation in number of clicks and the relatively small number of subjects in the SG group meant this was likely to be a type II statistical error. Body weight decreased significantly over the first year, but was stable between 1 to 6 years after the SG.

Visual analogue scales (VAS) were used to rate hunger, fullness, and desire to eat [23–26]. Accepting the limitations of the VAS, the rating of hunger, fullness feeling, prospective food intake, and nausea after the overnight fast remained similar over the 6 years in the SG group (*P* > 0.05). After the PRT the VAS measurements remained similar for hunger, sweetness, creaminess, liking and nausea over the 6 years.

The limitations of the study included the small sample size, albeit that the effect size of the surgery and the precision of the PRT were such that the study was adequately powered. The number of SG group participants decreased from 16 to 8, which is unfortunately expected considering the 6-year follow-up period [27]. However, we made sure to include data for the same participants in the SG group at all visits in order to ensure the consistency in the results. Participants in the comparator group were specifically recruited to have normal body weights to understand the breakpoints during the PRT in normal weight individuals. Despite the comparator group not being the same individuals as those studied 6 years previously, it is interesting to note that there was no difference in breakpoint between the comparator groups which was used at the start of the study and 6 years later.

Future studies will need to be larger to understand the magnitude of the changes in appetitive behaviour after bariatric surgery. It would also be important to have the same comparator group for all the time points to make sure there is no effect of learning on the progressive ratio task.

## Conclusion

Sleeve gastrectomy changed appetitive behaviour towards high fat and sugary candies over a period of 6 years. Understanding the mechanisms of weight loss and maintenance after bariatric surgery may help to explain to patients what they may expect but help improve safe and effective treatments for obesity.
